# Atypical Case of VV1 Creutzfeldt–Jakob Disease Subtype: Case Report

**DOI:** 10.3389/fneur.2022.875370

**Published:** 2022-05-09

**Authors:** Adrianna E. Carrasco, Brian S. Appleby, Ignazio Cali, Hamid R. Okhravi

**Affiliations:** ^1^School of Medicine, Eastern Virginia Medical School, Norfolk, VA, United States; ^2^Department of Neurology, Case Western Reserve University, Cleveland, OH, United States; ^3^Department of Pathology, Case Western Reserve University, Cleveland, OH, United States; ^4^Department of Internal Medicine, Glennan Center for Geriatrics and Gerontology, Eastern Virginia Medical School, Norfolk, VA, United States

**Keywords:** Creutzfeldt-Jakob disease, VV1 type, real-time quaking-induced conversion, dementia, Hashimoto's thyroiditis

## Abstract

Creutzfeldt–Jakob disease (CJD) is a rare form of rapidly progressive, neurodegenerative disease that results from the misfolding and accumulation of an aberrant, disease-associated prion protein (PrPD). CJD affects 1–1.5 cases per million per year with the sporadic-type accounting for an estimated 85% of these cases. Sporadic CJD (sCJD) is further subdivided into five subtypes based on genetic polymorphisms; the rarest subtype, sCJDVV1, occurs at a rate of 1 case per one-hundredth million population per year. Clinical characteristics of the sCJDVV1 subtype have been reported to show, early age of onset (44 years), average disease duration of 21 months, absent PSWCs on electroencephalography (EEG), and MRI hyperintensities in the cerebral cortex with usual negative signal in the basal ganglia or thalamus. We present a case of the sCJDVV1 subtype with uncommon features. Contrary to current data on sCJDVV1, our patient presented with an unusual age at onset (61 years) and longer disease duration (32 months). The highly sensitive and specific real-time quaking-induced conversion (RT-QuIC) assay was negative. Presenting clinical symptoms included paranoid thoughts and agitation, rapidly progressive memory decline, prosopagnosia, and late development of myoclonus and mutism. Other findings showed positive antithyroid peroxidase antibodies (anti-TPO), and absent PSWCs on EEG. High-dose steroid therapy treatment was administered based on positive anti-TPO findings, which failed to elicit any improvement and the patient continued to decline. To our knowledge, only four cases with the sCJDVV1 subtype, including our patient, have been reported to have a negative result on RT-QuIC. This may suggest varied sensitivity across sCJD subtypes. However, given the rarity of our patient's subtype, and the relatively novel RT-QuIC, current data are based on a small number of cases and larger cohorts of confirmed VV1 cases with RT-QuIC testing need to be reported.

## Introduction

Sporadic Creutzfeldt–Jakob disease (sCJD) is a rare, rapidly progressive, neurodegenerative disease that results from the misfolding and accumulation of an aberrant, disease-associated prion protein (PrP^D^) (1).

The pathogenic PrP isoform or PrP^D^ is the abnormal isoform of the naturally occurring cellular prion protein (PrP^C^). Compared to PrP^C^, PrP^D^ is highly enriched with insoluble ß-sheet structure, which confers to its characteristic of being partially resistant to enzymatic digestion by proteases (e.g., proteinase K, PK). PrP^D^ is believed to induce conformational changes in the physiological PrP^C^ which is, in turn, converted into PrP^D^ at an exponential rate, thus making this an essentially uncontrollable process (2, 3). The accumulation of PrP^D^ insoluble aggregates interferes with neuronal function and ultimately leads to cell death (3).

In the 1920s, Spielmeyer used the term “Creutzfeldt–Jakob disease” for the first time to describe a series of six unusual degenerative neuropathological cases previously described by Creutzfeldt and Jakob (4).

Modern classification of sCJD into five phenotypically distinct subtypes is based on the combination of two modifiers of the disease phenotype: (1) the type of the PK-resistant PrP^D^ (termed type 1 or type 2), (2) three possible genotypes at codon 129 of the PrP gene (129 met/met or 129 val/val homozygous, and 129 met/val heterozygous) (5, 6). This modern classification of sCJD has proven to be important as it facilitates the diagnosis of this group of prion diseases.

## Case Report

A 63-year-old Caucasian woman presented to ER with a 1-year history of behavioral disturbance and memory loss with more recent (2 months) episodes of agitation, delusional thoughts, questionable visual and auditory hallucinations, and aggressive behavior. In the ER, she appeared with elevated affect and was overly cheerful, with poor judgment and insight. Her speech was fluent and spontaneous but slightly increased in rate, rhythm, and volume. The rest of her physical exam was within normal limits without any abnormal neurological findings. She was admitted to the psychiatric unit for further assessment and management and was started on olanzapine for agitation and disturbing delusions. The electroencephalogram (EEG), CT head, and pertinent blood work were unremarkable. She was screened for cognitive impairment with the Montreal Cognitive Assessment and scored 19/30. She was discharged home after a one-week stay at the psychiatric unit with a diagnosis of major neurocognitive disorder associated with psychosis. She was seen in our memory clinic 2 months after discharge with a rapidly progressive cognitive and functional decline. She was dependent on most activities of daily living, such as bathing, grooming, and dressing, and became socially withdrawn with reports of prosopagnosia in the last several months. Her neuropsychological testing assessment showed severe impairment in memory, attention, executive function, language, and visuospatial domains. She scored 7/30 on Mini-Mental State Examination. Her neurological examination remained unremarkable, except for motor apraxia. She underwent extensive workup for rapidly progressive dementia.

**Figure 1:**
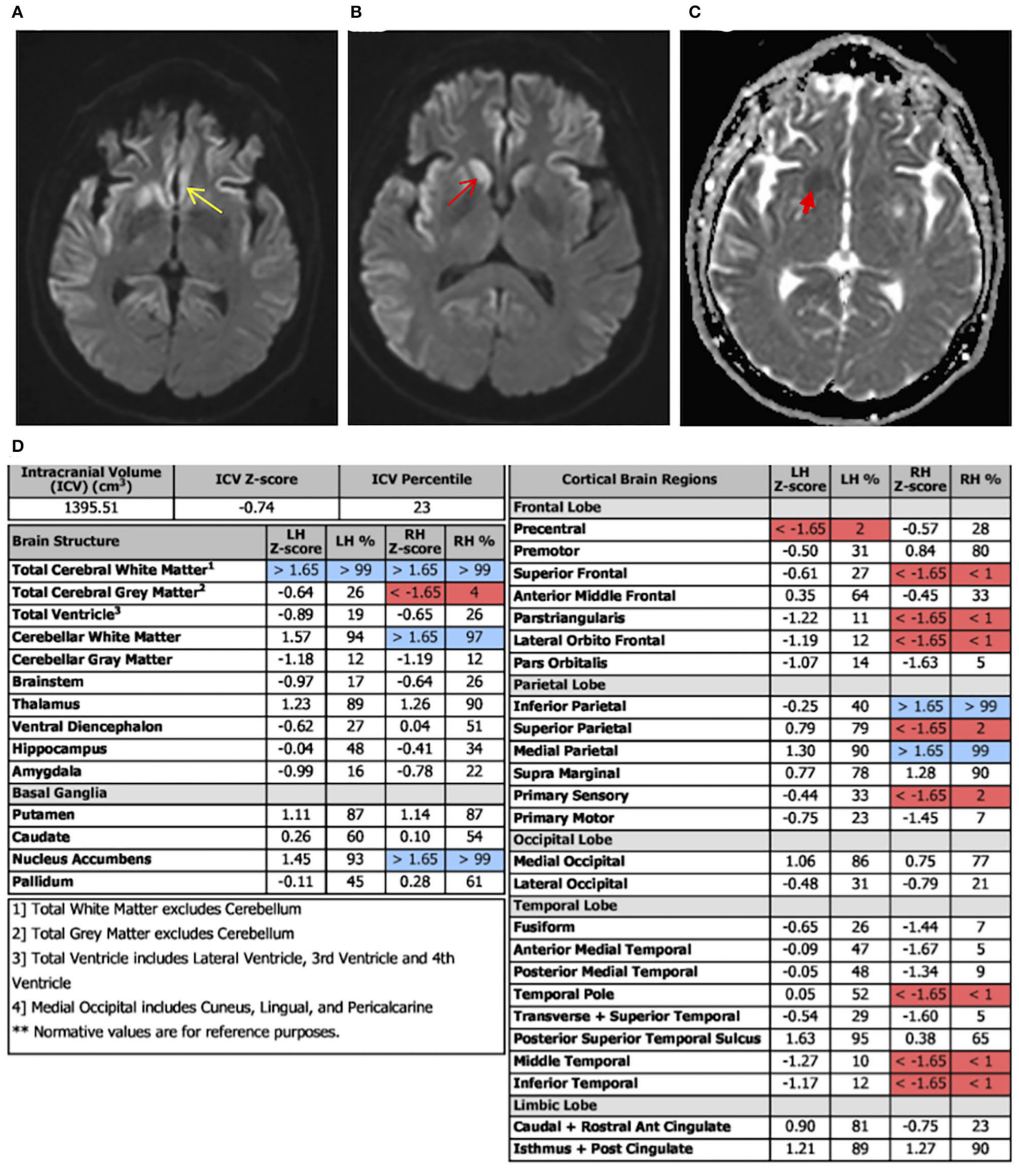
Case report timeline.

Laboratory tests performed at this time included paraneoplastic profile, serum, and urine heavy metals, infectious processes such as HIV, syphilis, and Lyme disease, which were found to be negative; however, she had both elevated antithyroid peroxidase (TPO-Ab) and antithyroglobulin (TG-Ab) antibodies indicative of Hashimoto's thyroiditis. An MRI of the brain with volumetric studies was performed. The MRI showed asymmetric (right>left) diffusion-weighted imaging (DWI) signal hyperintensities in the cortical ribbon of the parietal, frontal, and temporal lobes, hippocampi, caudate, and putamen (Figures 1A,B). There were no signal abnormalities in the perirolandic gyri, occipital cortex, thalami, and cerebellum. This pattern was highly suggestive of CJD. Volumetric studies showed mainly right-sided frontal, parietal, and temporal lobes atrophy. Hippocampal and medial temporal lobe volumes were within normal limits.

**Figure 2:**
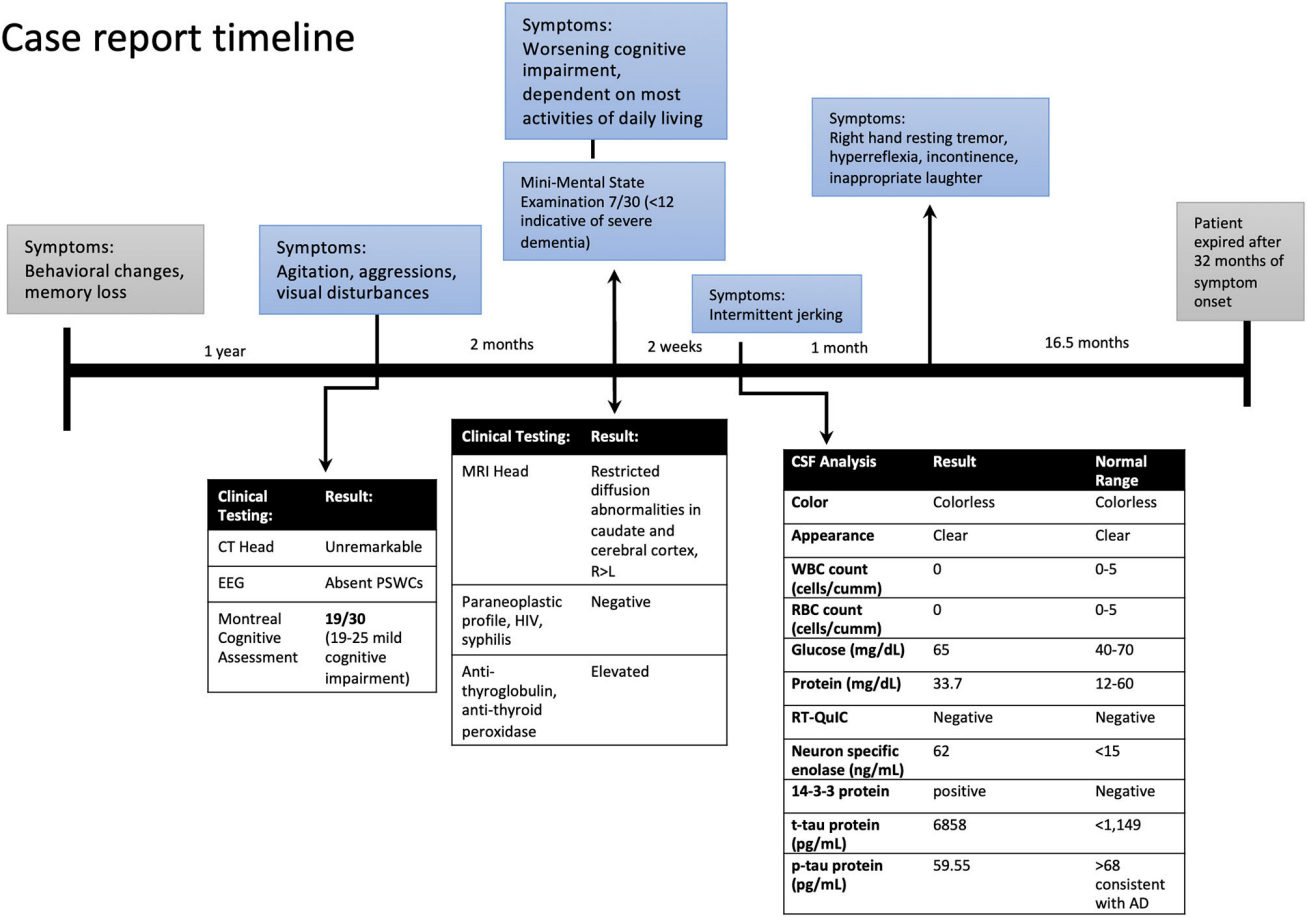
Diffusion-weighted imaging (DWI) axial view, apparent diffusion coefficient (ADC) view, and regional volumetric studies. Asymmetric DWI increased signal intensity in the cortical ribbon of the parietal, frontal, and temporal lobes, hippocampi, caudate, and putamen. The signal hyperintensities are greater on the right. **(A)** Image shows increased signal intensity in the cingulate cortex (yellow *arrow)*. **(B)** More superior image shows diffuse asymmetric involvement of parietal, frontal, and temporal lobes. There is notable involvement of the caudate head that is greater on the right (red *arrow*). **(C)** ADC sequence illustrating caudate changes (red *arrow*) consistent with the DWI hyperintensities. **(D)** Brain atrophy pattern (highlighted in red), mainly on the right frontal, temporal, and parietal lobes, consistent with areas of abnormal DWI in MRI.

At her follow-up visit 2 weeks later, the husband reported the patient developed intermittent jerking movements in her extremities. The patient underwent a lumbar puncture and repeat EEG. The cerebrospinal fluid (CSF) analysis showed no RBCs, elevated 14-3-3 protein, elevated t-tau protein levels of 6,858 pg/ml (normal range <1,149), amyloid-beta 42 level of 185.85 pg/ml (normal range >1,026 pg/ml) and p-tau of 59.55 pg/ml (normal <54 pg/ml). The t-tau/p-tau ratio was 115. Neuron-specific enolase (NSE) was also elevated at 62 ng/ml (normal range <15). Repeat EEG did not show periodic sharp wave complexes (PSWCs). A real-time quaking-induced conversion (RT-QuIC) test was negative. On her follow-up visit she had developed new symptoms; a resting tremor of the right hand, biceps tendon hyperreflexia, inappropriate laughter, urine and stool incontinence, and began eating nonfood items. Two months from her initial memory consultation visit, her exam was significant for myoclonic jerks and evolving mutism, highly suspicious for probable CJD. She also received 3 courses of high-dose steroid therapy followed by a tapering dose of prednisone for the possibility of Hashimoto's encephalopathy. The patient failed to elicit any improvement in steroid treatment, continued to decline, and was transitioned to hospice care. The patient's decline continued, requiring total care. She became bedbound and the patient expired 20 months after the CJD diagnosis, and her brain was sent to the National Prion Disease Pathology Surveillance Center (NPDPSC) for autopsy. The illness duration from symptom onset (neuropsychiatric symptoms) to death was 32 months (Figure 2). The long duration of the symptoms is consistent with the typically prolonged survival of VV1 (7).

**Figure 3:**
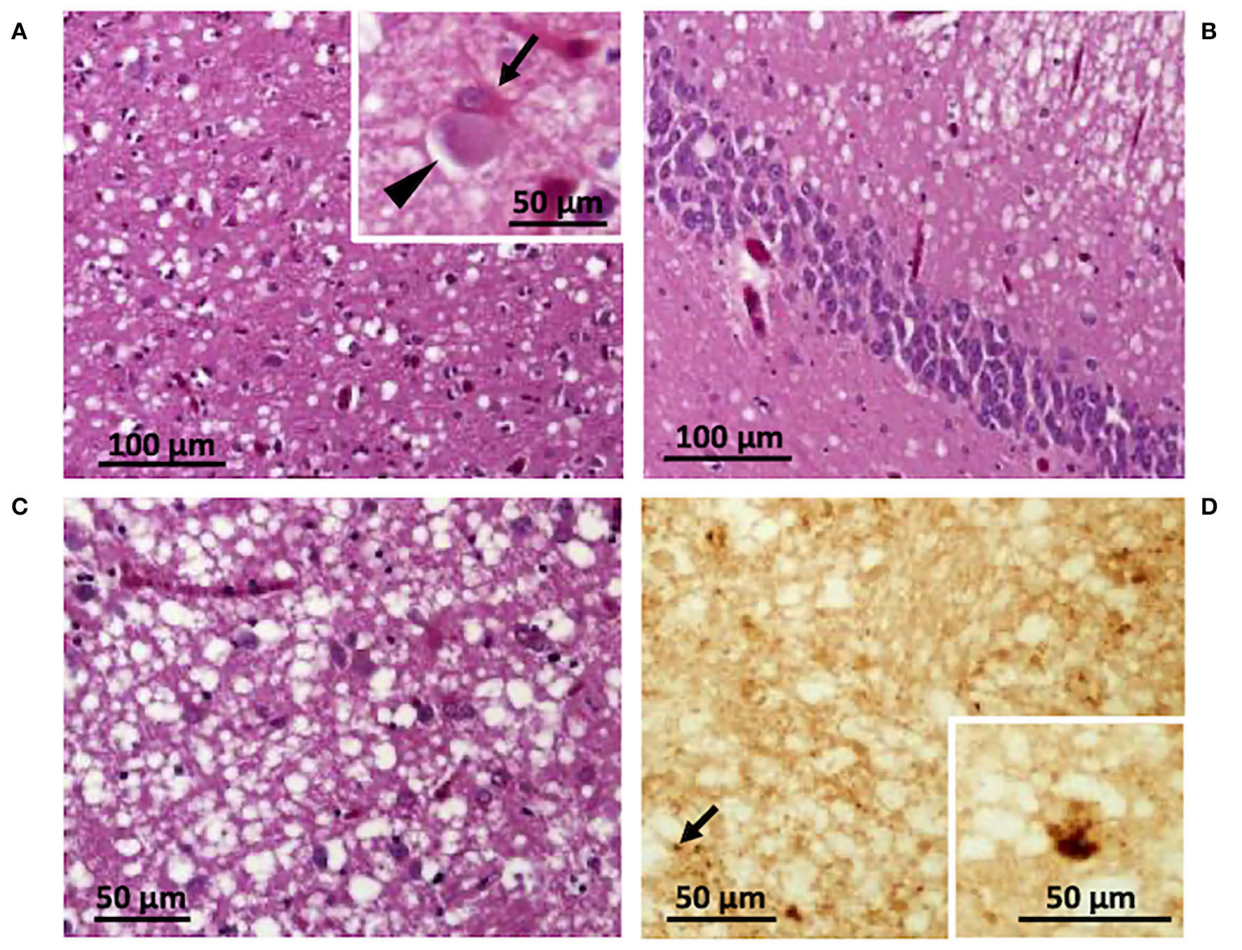
Histological and immunohistochemical features. Spongiform degeneration with intermediate size vacuoles affecting cortical **(A,B)** and subcortical regions **(C)**; inset in **(A)** a ballooned neuron (*arrowhead*) and a reactive astrocyte (*arrow*). **(D)** Immunohistochemistry showing PrP granules (*arrow*) and dispersed deposits of larger PrP aggregates (inset). **(A)** Parietal cortex; **(B)** hippocampus; **(C,D)** putamen. Antibody: 3F4.

The autopsy included histopathological and immunohistochemical analysis, western blot, and genetic testing. Western blot findings demonstrated PK-resistant PrP^D^ type 1, with the unglycosylated PrP^D^ isoform migrating to ~ 20 kDa (data not shown) (7), and histopathological features of the VV1 subtype (Figure 3) (8). Genetic analyses did not detect pathogenic mutations of the prion protein gene (*PRNP*) and the patient was valine homozygous at codon 129 (129VV). The final diagnosis was sporadic CJD of the VV1 subtype (6, 7).

## Background (Etiology, Pathology/Pathophysiology)

### Classification, Epidemiology, and Clinical Course

Creutzfeldt–Jakob disease (CJD) affects 1–1.5 cases per million per year and is classified into three types: sporadic (sCJD), genetic, or acquired (1). Acquired forms of the disease, which include kuru, iatrogenic, and variant CJD, account for <1% of all cases (8). The genetic form, which accounts for 5–15% of cases, is due to an autosomal dominant mutation in the prion protein gene, *PRNP*.

Sporadic CJD (sCJD) is the most commonly occurring human prion disease, as it accounts for 85% of all cases. sCJD is further divided into five subtypes based on the type of PrP^D^ (types 1 and 2) and polymorphisms at codon 129 of the PrP gene (129 MM, MV, or VV) (9–11). Based on the combination of the aforementioned molecular features (PrP^D^ type and codon-129 genotype) sCJD includes the following subtypes: (1) MM/MV1; (2) VV1; (3) MM2; (4) MV2; and (5) VV2. The MM1 and MV1 groups were combined into one subtype (MM/MV1) because of the virtually identical phenotype. While sCJDMM1 is the most common human prion disease, the sCJDVV1 represents the rarest subtype, as it occurs at a rate of 1 case per one-hundredth million population per year (12). The mean age at onset of sCJDMM1 is 66 years, with a disease duration of 4 months. Unlike sCJDMM1, clinical characteristics of the rare VV1 subtype have been reported to show, early age of onset (44 years), disease duration of 21 months, elevated 14-3-3 and total tau in the CSF, absent PSWCs on electroencephalography (EEG), and magnetic resonance imaging (MRI) hyperintensities in the cerebral cortex with usual negative signal in the basal ganglia or thalamus (9–11). CSF RT-QuIC is typically negative in VV1 cases (13). The prodromal phase in VV1 cases also differs from typical sCJD in that it begins as slowly progressive dementia with behavioral symptoms for an extended time (7 months) in contrast to initial cognitive decline followed by increased neurologic symptoms within weeks (12).

### Diagnostic Criteria for CJD

The Centers for Disease Control (CDC) and WHO have established guidelines for the classification of probable, possible, and definite sCJD based on neuropathology or specific combinations of antemortem clinical testing and symptoms (1, 14, 15). Currently, it is only possible to make the definitive diagnosis of CJD through postmortem analyses of cerebral tissue. The diagnosis requires the presence of spongiform vacuolation, gliosis, neuronal loss, and PrP immunostaining (1). The well-known classical symptoms associated with sCJD include rapidly progressive dementia, ataxia, myoclonus/spasticity, and PSWCs on EEG (15). However, this combination of symptoms may not be present at every stage of the disease or at all in some cases (1, 8, 13). Thus, clinical testing and findings for probable sCJD must meet the subsequent conditions: PSWCs on EEG, one of the following imaging requirements; high signal in caudate/putamen on brain MRI, at least two cortical regions (frontal, temporal, parietal, and occipital) on either DWI or fluid-attenuated inversion recovery (FLAIR), and laboratory results showing increased levels of biomarkers in CSF and positive RT-QuIC results (1, 13, 15).

### EEG and Imaging Findings

The presence of PSWCs on EEG is a highly recognized feature of CJD. This finding carries a 65% sensitivity and a specificity between 74 and 86%, however, PSWCs are not sufficient to make a CJD diagnosis (6, 13, 15–17). Furthermore, not all subtypes of CJD will demonstrate the same EEG abnormalities. For example, MM1 and MV1 display PSWCs' EEG waveforms while 42% of MM2 cases have PSWCs present, and VV1 does not (10, 12, 18). Advances in MRI techniques, such as FLAIR and DWI, have allowed physicians to improve the negative and positive predictive value in diagnosing CJD. Based on a recently published study, the sensitivity of MRI in aiding in the detection of sCJD is 90 and 95% with a specificity of 90 and 100% (19). Areas of gliosis and vacuole formation, associated with the disease process, have been found to correlate with high-intensity signal patterns on T2/Flair and DWI, respectively (20, 21). Our case underwent two separate EEG evaluations, both of which failed to demonstrate PSWCs. A study by Geschwind et al. (20) looked at the significance of tracking disease progression through hippocampal volume and other regions of interest in sCJD cases *via* various imaging techniques. This study found a discordance among imaging assessments' regional involvement for sCJD cases, especially within the frontal and anterior and mid-limbic areas. Recent studies have described sCJD DWI hyperintensities to fluctuate based on disease stage, following a “J” or “U-shaped” curve (22, 23). According to this principle, diffusivity on MRI may be present in the early stages without any atrophy patterns among the same regions. Furthermore, as the disease process continued, DWI hyperintensities would fluctuate, yet brain atrophy would emerge and advance throughout the disease. This suggests the importance of correlating volumetric changes, which are progressive, with MRI findings for proper disease staging. Interestingly, our case had normal hippocampal volumes in volumetric studies and showed hyperintensity involvement in DWI, which may indicate an early time point in our patient's disease course. However, counter to the hippocampal comparisons, there were correlations in the cortical regions between DWI and volumetric studies (Figures 1A–C).

### CSF Biomarkers

Cerebrospinal fluid (CSF) biomarkers that are commonly used to help establish a CJD diagnosis include, 14-3-3 protein and tau-protein. A systematic review showed that the 14-3-3 protein has a sensitivity and specificity of 83.3 and 78% in identifying sCJD respectively (24). The tau protein has sensitivity and specificity ranging from 75 to 100% and 49 to 100% (25, 26). However, these biomarkers demonstrate a varied sensitivity that is dependent on the 129 codon polymorphism with the VV1 carrying the highest sensitivity, approaching 100% (10, 16, 17). Emerging data on the diagnostic utility of neurofilament light chain (NfL), (a surrogate biomarker of neuroaxonal degeneration), have indicated a possible role as a highly sensitive, supportive third-level biomarker in prion disease cases with atypical clinical and laboratory findings (27–29). Compared to patients with Alzheimer's disease, CSF NfL has been reported to be significantly higher in patients with prion disease and may have characteristic subtype levels (27). There is currently limited data on CSF NfL levels for sCJDVV1, however, several studies have reported a handful of cases of this subtype to yield the highest levels among their study cohorts (27–29). Furthermore, given that these markers are released with neuronal damage and found in other neurodegenerative diseases, positive CSF results for NfL, 14-3-3, and/or tau protein are nonspecific for CJD.

Since its introduction in 2010, the RT-QuIC assay is quickly becoming the preferred method of detecting PrP^Sc^ in suspected CJD cases (30). RT-QuIC exploits the PrP^Sc^ self-replicating ability and aggregate formation, thus yielding a highly sensitive and specific test (30, 31). Modifications in the RT-QuIC assay have been made and improved its sensitivity. According to a recent analysis out of a large prion disease pathology surveillance center, RT-QuIC diagnostic sensitivity and specificity were found to be 90.3% and 98.5% for all prion disease types (13). The study also noted a decreased sensitivity in familial CJD (fatal familial insomnia, Gerstmann–Sraussler–Scheinker disease, and sporadic fatal insomnia) and the VV1 and MM2 sCJD subtypes. The latter findings are agreeable with our case's negative RT-QuIC.

## Discussion

Classical features of sporadic prion disease such as the age of onset, clinical presentation, diagnostic findings, progression, and average disease length, have largely been characterized but with less frequency in the rarer sCJDVV1 subtype (Table 1) (7). As stated earlier, this subtype has been described to have an average age of onset of 44 years and progressive dementia of the frontotemporal type which may develop for months without any other neurological findings (8, 11, 12, 32). Our patient was 61 years of age when they had first developed symptoms, which is an older age of onset for sCJDVV1. A notable discrepancy in our case was her length of the disease. The initial reported neuropsychiatric symptoms, namely, anxiety and mood disturbances, occurred one year before the first hospital admission, thus resulting in a total disease course of 32 months.

**Table 1:** Comparison of typical VV1 characteristics and our case*.

**Characteristic**	**Our case**	**Typical VV1**
**Subject demographic, disease course**		
Sex	Female	Male predominance
Age of onset (years)	61	44 (19–55)
Disease duration (months)	32	21 (17–49)
**Clinical features**		
Cognitive problems^a^	Yes	Yes
Aphasia	Yes	Yes
Apraxia	Yes	Occasionally
Visual disturbances^b^	Yes, early	Occasionally
Limb or gait ataxia	No	Yes
Myoclonus	Yes	Yes
Pyramidal symptoms^c^	Yes, rigidity	Yes
Psychiatric symptoms^d^	Yes, early, agitation, aggressions	Yes, regression, fear, aggressions
**EEG**		
PSWCs^e^	No	No
**MRI**		
Widespread cortical signal involvement	Yes, right predominance	Yes
Basal ganglia signal increase	Yes, caudate and putamen	Sometimes
**Laboratory findings**		
14-3-3 positive	Yes	Yes
Tau	Yes, significantly elevated	Yes, significantly elevated
RT-QuIC	Negative	Unknown -mostly negative
**Neuropathological features**	Spongiform degeneration affecting cortical and subcortical regions	Severe spongiform changes in the cerebral cortex and striatum

*Typical characteristics based on published findings from Meissner et al. (12).

*^a^Memory loss, dementia, and confusion*.

*^b^Visual loss, field defect, blindness, and distortion*.

*^c^Rigidity or spasticity*.

*^d^Anxiety, depression, aggression, psychosis, personality changes, and regression*.

*^e^PSWCs – periodic sharp wave complexes on electroencephalography*.

Given the combination of a relatively new test for a rare disease and an even rarer subtype, we found four cases of VV1, including ours with a negative RT-QuIC (33). In a recent large study in the United States which analyzed the diagnostic accuracy of RT-QuIC, all of the VV1 (*n* = 3) cases were shown to be negative (13). Another study by Lattanzio et al. (34) investigated the diagnostic value of prion RT-QuIC in pathologically confirmed cases and described a diverse sensitivity reliant on the codon 129 genotype. For example, they found RT-QuIC sensitivity to be higher in MM (84.2%) than in either MV (72.2%) or VV (79.5%) cases, with the more common VV2 type contributing to this percentage. Additional studies investigating similar parameters also indicated the RT-QuIC's varied sensitivity across sCJD subtypes, especially in the VV1, and MM2-C forms (35–37). These strongly suggest that the diagnostic advantage of RT-QuIC may be related to the type of prion strain present, thus demonstrating a possible concern in the CDC's updated guidelines for CJD. The RT-QuIC analysis was first used clinically in 2015 in the United States. Following extensive supportive research, it was included in the CDC's 2018 most recent updated clinical diagnostic criteria guidelines for a probable diagnosis of sCJD (1, 14). Although this relatively new clinical test has been proven to be highly sensitive for the common subtype, these recent findings indicate a disproportionate negative RT-QuIC result for the MM2 and especially the VV1 subtypes. Further investigations of pathologically confirmed VV1 cases combined with RT-QuIC testing need to be reported to establish this unique characteristic. In a different study, brain MRI and CSF RT-QuIC had a combined sensitivity of 100% for all sCJD subtypes (19).

Recently, MRI, FLAIR, and DWI have increased their utility in early CJD detection. For example, classical sCJD changes on MRI such as putamen and caudate head hyperintensities may present before anticipated EEG findings (38). In sCJD VV1 hyperintensities are found in the cerebral cortex, sometimes in the basal ganglia, and are commonly lacking within the thalamus (10, 12, 39). Our patient demonstrated asymmetric DWI signal hyperintensities in the cortical ribbon of the parietal, frontal, and temporal lobes, basal ganglia, and hippocampi, which are consistent with VV1.

Pathological features of the VV1 include involvement of the corticostriatal regions and relative sparing of the occipital lobe compared to the frontal and temporal lobes (7, 10). Our case demonstrated these features. Furthermore, spongiform degeneration with intermediate size vacuoles affecting cortical and subcortical regions and ballooned neurons, two typical pathological features of the VV1 subtype, were present in our case (Figures 3A–C). Immunohistochemistry revealed a faint PrP staining and occasionally larger PrP aggregates (Figure 3D).

As anticipated, this pattern correlated with the signal hyperintensity findings on DWI. While CJD is currently an uncurable disease, these correlations may be useful in the antemortem setting for symptom management, disease pattern recognition, and insight into the disease microenvironment and cerebral region susceptibility.

## Treatment Considerations

Despite numerous clinical trials, there are currently no effective treatments for CJD. Four drugs, flupirtine, quinacrine, pentosan polysulfate (PPS), and doxycycline have been studied on a large scale and are directed toward inhibiting the conversion of PrP^c^ to PrP^sc^ (40). Another emerging treatment approach for prion disease has been aimed at reducing the expression of PrP^c^ *via* antisense oligonucleotides (ASOs) (41). These PrP^c^ lowering therapies have yielded promising results in mice models across five different CJD subtypes (42). A recent study found that a CSF bolus of ASOs in prion-infected mice extended survival time by 61–98% (41). However, the effective dosing regimen and degree of adverse side effects of ASOs in human trials are currently unknown, and emerging clinical trials using this treatment modality are likely to take place in the near future.

At this time care for highly suspected cases is predominately aimed at symptom management and advanced care planning with families/next of kin. Regardless of ineffective clinical trials, it is important to identify potential sCJD cases early to provide adequate time for families to care for their loved ones and for those who wish to decide on study participation (40).

## Conclusion

Our patient was a complex case in the sense that she demonstrated findings that differ from the other more commonly occurring forms of sCJD. The EEG findings and extended prodrome did not follow patterns of typical sCJD and the patient's age was older than most VV1 cases. Furthermore, studies indicate the probability of PSWCs on EEG increases with increasing age, and subtype, and is dependent on the disease stage (32). These unclear diagnostic findings paired with negative RT-QuIC results and elevated anti-TPO levels manifested a complex clinical judgment call for the care team and family. It is possible sCJDVV1 cases with a presentation similar to ours (older age, lengthy cognitive, and/or psychiatric prodrome without clear etiology) may go undiagnosed, especially if nonstandard diagnostic tests, such as CT, instead of MRI are performed. The presentation of rapidly progressive cognitive and functional decline, in conjunction with the late-adult onset of psychiatric disturbances in patients without any obvious infectious, malignant, or traumatic etiologies, should have sCJD included in their differential. Early suspicion of prion disease is important for proper management of tissue and specimens, adequate advanced care planning for the patient and loved ones, and to enable sufficient time to make an end of life decisions for their next of kin, or adequate time to undergo clinical trials. Potential future therapies may also require subtype classification given the isoforms' theorized unique molecular interactions or their role in disrupting vital neuronal pathways and requiring different outcome measures given the clinical heterogeneity between sCJD subtypes. Furthermore, patients with a high suspicion of sCJD and negative RT-QuIC results should consider VV1, and other atypical prion diseases (e.g., sporadic fatal insomnia, variably protease-sensitive prionopathy, and some genetic forms of prion disease).

## Data Availability Statement

The original contributions presented in the study are included in the article/supplementary material, further inquiries can be directed to the corresponding author.

## Author Contributions

AC wrote the manuscript with support from HO, BA, and IC. AC and HO contributed to conception and design of the report. HO wrote the case report section. BA contributed to the background, discussion, treatment considerations, and conclusion sections of the manuscript. IC wrote the description of Figure 1 and contributed to the background section of the manuscript. BA and IC provided immunohistochemistry sections. All authors contributed to manuscript revision, read and approved the submitted version.

## Funding

This study was in part supported by the NIH NIA K99 AG068359 to IC.

## Conflict of Interest

The authors declare that the research was conducted in the absence of any commercial or financial relationships that could be construed as a potential conflict of interest.

## Publisher's Note

All claims expressed in this article are solely those of the authors and do not necessarily represent those of their affiliated organizations, or those of the publisher, the editors and the reviewers. Any product that may be evaluated in this article, or claim that may be made by its manufacturer, is not guaranteed or endorsed by the publisher.
